# Supplementary material for: Sodium Selenite Alleviates Breast Cancer-Related Lymphedema Independent of Antioxidant Defense System
Source: Nutrients. 2019 May 7;11(5):1021. doi: 10.3390/nu11051021 (PMC6566195; doi:10.3390/nu11051021)
Supplement: Supplementary file 1 [file nutrients-11-01021-s001.pdf]

Table S1. Description of intervention schedule.

Session	Time from first session (days)			p
	**Control (*n* = 12)**	**Selenium (*n* = 14)**	**Total (*n* = 26)**	
Second session	3.3 ± 0.8	3.0 ± 1.1	3.2 ± 1.0	0.560
Third session	6.9 ± 1.9	6.4 ± 2.1	6.6 ± 2.0	0.432
Fourth session	9.8 ± 2.4	9.9 ± 3.7	9.8 ± 3.1	0.667
Fifth session	13 ± 2.8	13 ± 5.1	13 ± 4.2	0.940

Values are presented as mean ± standard deviation (SD). Significant differences between groups were determined by Mann–Whitney *U* test.

Table S2. Proportion of patients by lymphedema stage.

	**CTRL (*n* = 12)**			**SE (*n* = 14)**		
	Baseline	2-week	Follow-up	Baseline	2-week	Follow-up
Stage Ⅱ	1 (8.33)	1 (8.33)	2 (16.7)	2 (14.3)	11 (78.6)	12 (85.7)
Stage Ⅲ	11 (91.7)	11 (91.7)	10 (83.3)	12 (85.7)	3 (21.4)	2 (14.3)
p *		0.309			0.001	
p †			0.002			

Values are presented as *n* (%). * *p*-values were determined by generalizing estimating equation using time as a fixed effect. † *p*-values were determined by generalizing estimating equation using time × group as a fixed effect.
